# Ensuring Gluten-Free Safety: A Descriptive Analysis of Laboratory Results and Quality Control

**DOI:** 10.3390/foods15071144

**Published:** 2026-03-26

**Authors:** Roberta Giugliano, Laura Migone, Bianca Saccheggiani, Simona Mella, Elisabetta Razzuoli

**Affiliations:** National Reference Centre for the Detection of Substances and Products Causing Allergies or Intolerances in Food (CReNaRiA), Istituto Zooprofilattico Sperimentale del Piemonte, Liguria e Valle D’Aosta, Piazza Borgo Pila 39/24, 16129 Genoa, Italy; laura.migone@izsplv.it (L.M.); bianca.saccheggiani@izsplv.it (B.S.); simona.mella@izsplv.it (S.M.); elisabetta.razzuoli@izsplv.it (E.R.)

**Keywords:** gluten-free, food safety, food control

## Abstract

Ensuring the safety of gluten-free foods is essential for individuals with coeliac disease and other gluten-related disorders, for whom even minimal gluten exposure can cause adverse effects; this study aimed to evaluate the long-term compliance of gluten-free labeled foods marketed in Italy. A total of 4139 pre-packaged gluten-free products were collected between 2015 and 2024 and analyzed using validated analytical methods. Products were categorized into macro-categories: cereal-based foods, processed non-cereal-based foods, confectionery, flours, baby foods, and dietary supplements. A descriptive analysis and risk modeling were generated to visualize relative risks. Overall non-compliance remained consistently very low (<1%) throughout the 10-year period, with an average rate of 0.27% and minor peaks in 2016 and 2018. The highest frequencies of gluten contamination were observed in cereal-based products and flours-particularly corn flour-while occasional non-compliance occurred in some processed non-cereal-based foods and confectionery; no non-compliance was detected in baby foods or dietary supplements. These findings are reassuring and consistent with, or better than, available EU data, confirming the effectiveness of current control systems and highlighting the importance of continuous monitoring, validated analytical methods and effective allergen management strategies. Strengthened collaboration among regulators and manufacturers remains essential to prevent cross-contamination and protect consumer health.

## 1. Introduction

Gluten, a protein found in wheat, barley, and rye, is a common component of many staple foods. However, for some individuals, consuming gluten can lead to adverse health reactions. These gluten-related disorders differ in clinical presentation, severity, and underlying pathogenic mechanisms [1,2,3,4,5,6,7,8,9,10,11,12,13,14,15,16].

The most well-known of these conditions is coeliac disease (CD), an autoimmune disorder in which gluten ingestion triggers an immune-mediated inflammatory response that causes the small intestine mucosal damage, malabsorption and both gastrointestinal and systemic symptoms [1,2,3,4,5,7,14,17]. CD affects approximately 1% of the population and requires lifelong adherence to a gluten-free diet [5,7,9,10,11,12].

Another condition is non-coeliac gluten sensitivity/wheat sensitivity (NCGS/WS), characterized by symptoms like CD (e.g., bloating, fatigue, and abdominal pain) but without evidence of autoimmune intestinal damage or serological markers [1]. Although its mechanisms remain unclear, NCGS/WS can significantly impact quality of life. Additionally, wheat allergy is an IgE-mediated immune response to proteins found in wheat, including gluten, other wheat components such as amylase/trypsin inhibitors and fermentable oligosaccharides, disaccharides, monosaccharides, and polyols [1].

Epidemiological data indicate increasing CD diagnoses over recent decades in several countries, including Italy [8,9,10,18]. In Italy, prevalence is estimated at around 1.15% [18,19,20,21].

According to the Codex Alimentarius, gluten-free foods are dietary foods: (a) consisting of or made only from one or more ingredients which do not contain wheat (i.e., all Triticum species, such as durum wheat, spelt, and khorasan wheat, commercially marketed under the name such as KAMUT^®^), rye, barley, oats or their crossbred varieties, and the gluten level does not exceed 20 mg/kg in total, based on the food as sold or distributed to the consumer, and/or (b) consisting of one or more ingredients from wheat which have been specially processed to remove gluten, and the gluten level does not exceed 20 mg/kg in total, based on the food as sold or distributed to the consumer [8,9,10].

Packaged food products bearing the “Crossed Grain” registered Trademark (TM) comply with the regulatory limit for gluten content (not exceeding 20 mg/kg), and additionally comply with stringent production, management, and process-control requirements outlined in the AOECS Standard for gluten-free foods (Version 3.0, 2022) [17]. This trademark is widely recognized in Europe and beyond, supported by harmonized licensing procedures coordinated among national coeliac associations (https://www.celiachia.it/; https://www.aoecs.org/; https://www.coeliac.org.uk/, accessed on 20 January 2026).

European regulations—including Regulation (EU) 1169/2011 and Regulation (EU) 828/2014—define allergen-labeling obligations and specify the conditions for the use of gluten-free claims (≤20 ppm gluten). Conversely, Precautionary Allergen Labeling (PAL) remains voluntary, as the implementing acts of Regulation 1169/2011 have not yet been issued.

Although several international studies have investigated gluten contamination in processed foods and gluten-free products, peer-reviewed research specifically focused on the Italian market remains limited, particularly regarding multi-year datasets derived from official control activities [5,7,9,10,11,12,18,20]. In this regulatory and epidemiological context, the present study focuses specifically on Italian data collaborating directly with the Italian Coeliac Association (AIC), resulting in access to a uniquely comprehensive and systematically collected national dataset.

At present, harmonized, high-resolution datasets covering the entire European Union are not publicly available, and differences in sampling strategies, analytical reporting, and data accessibility limit the feasibility of conducting an EU-wide analysis with sufficient statistical robustness. Therefore, this work represents an initial, data-driven assessment based on the high-quality information available for Italy, while laying the foundation for broader comparative studies as more extensive and harmonized European datasets become available.

Accordingly, the aim of this study is to provide an evidence-based assessment of gluten occurrence in foods marketed in Italy, to evaluate compliance with EU Regulation 828/2014 across food macro-categories and sample types, and to identify matrices with relatively higher risk of non-compliance. By addressing these objectives, the study contributes essential and currently underrepresented data to the scientific literature on gluten-free product reliability.

## 2. Materials and Methods

### 2.1. Sampling and Data Source

The dataset analyzed in this study was obtained from the internal management system of the National Reference Centre for the Detection of Substances and Products Causing Allergies or Intolerances in Food (CReNaRiA), which archives all samples analyzed within official control activities and their corresponding laboratory reports. Each record in the database represents a single sample analyzed for gluten content. For each sample, the following variables were available: (i) sample type, (ii) analytical outcome (compliant or non-compliant), and (iii) year of analysis.

The study period covered ten years, from 2015 to 2024.

### 2.2. Sample Classification

Following data extraction, all samples were assigned to one of six main macro-categories, based on their composition and intended use:

Baby Food

Products formulated for infants and young children, including fruit-, vegetable-, meat-, dairy-, and cereal-based baby foods, as well as infant beverages such as chamomile infusions.

Flours

Including cereal (e.g., rice, corn, oat, millet) and pseudocereal flours (e.g., amaranth, quinoa, buckwheat), as well as mixed flours.

Processed Non-Cereal-Based Products

A broad category comprising legume-based powders (e.g., chickpea, soy, pea), nut- and seed-based products, tuber-derived preparations (e.g., potato and tapioca starches), cocoa- or chocolate-based products, plant-based beverages, additives (e.g., colorings, flavorings, gelling agents), vegetable-based preparations, and other processed foods not primarily derived from cereals.

Cereal-Based Products

Products where cereals or pseudocereals are a main ingredient or structural base, including bread, biscuits, cakes, pasta, cereal drinks, and ready-to-eat cereal products. Pseudocereal-based items (e.g., quinoa or buckwheat products) were included when their structure or technological function resembled cereal-based foods.

Confectionery and Sweets

High-sugar and/or high-fat products such as candies, chocolate bars, spreads (e.g., chocolate, peanut, vanilla), syrups, and sugar/sugar-paste/sweetener products.

Dietary Supplements

Products intended to supplement the diet, including vitamins, minerals, botanicals, amino acids, and other concentrated nutrient preparations, are marketed in capsule, tablet, powder, or liquid form.

For each sample, the analytical outcome was classified as: Compliant (C): gluten concentration ≤ 20 mg/kg; Non-compliant (NC): gluten concentration > 20 mg/kg, in accordance with EU Regulation 828/2014.

The final dataset consisted of 4139 samples.

### 2.3. Laboratory Analysis

The analyses were conducted exclusively within our laboratories, following an internal procedure with a method developed, validated, and accredited by the CReNaRiA.

Gluten determination was performed in our laboratories using a validated and accredited internal method based on the RIDASCREEN^®^ Gliadin R5 ELISA (R-Biopharm AG, Darmstadt, Germany), recognized as the Codex Alimentarius Type I reference method for gluten analysis and approved as AOAC OMA 2012.01. Samples were extracted using the patented Cocktail buffer following the Méndez protocol [22]. The assay employs the R5 monoclonal antibody, specific for immunotoxic epitopes from wheat, rye, and barley, with no cross-reactivity to oat avenins. The method has an LOD of 2.5 mg/kg and an LOQ of 5 mg/kg. All measurements were performed according to the manufacturer’s instructions using standard laboratory equipment, in detail, the SUNRISE, TECAN (Switzerland) microplate reader, the 715 laboratory shaker (ASAL, Milan, Italy) and the IPP 110 thermostat incubator (ENCO, Venezia, Italy).

The method has a Limit of Detection (LOD) of 2.5 mg/kg and a Limit of Quantification (LOQ) of 5 mg/kg.

### 2.4. Data Cleaning and Preparation

Prior to analysis, a data-quality assessment was performed to ensure completeness and consistency. The following steps were applied: removal of internal laboratory controls, as they did not correspond to market samples and were not relevant for regulatory assessment; verification of missing or duplicate entries (none retained); and consolidation of sample-type names to ensure consistent categorization across years.

After cleaning, the dataset was prepared for statistical evaluation by aggregating counts per macro-category, sample type, and year.

### 2.5. Statistical Analysis

**Figure 1:**
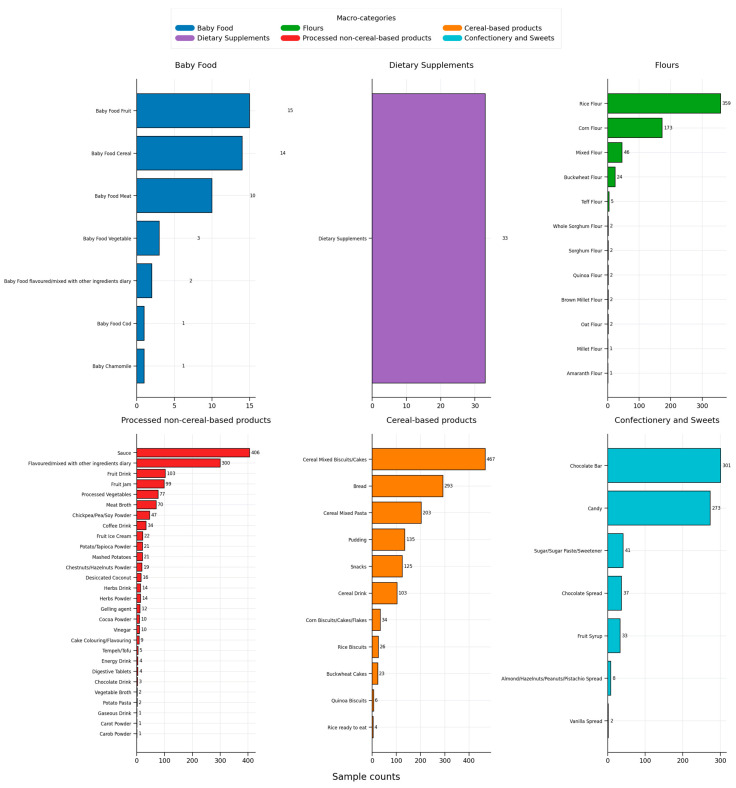
Total number of samples by sample-type within each macro-category (absolute counts on bars).

Descriptive statistics were used to summarize the distribution of sample counts across categories. Absolute frequencies were visualized using bar charts (Figure 1).

**Figure 2:**
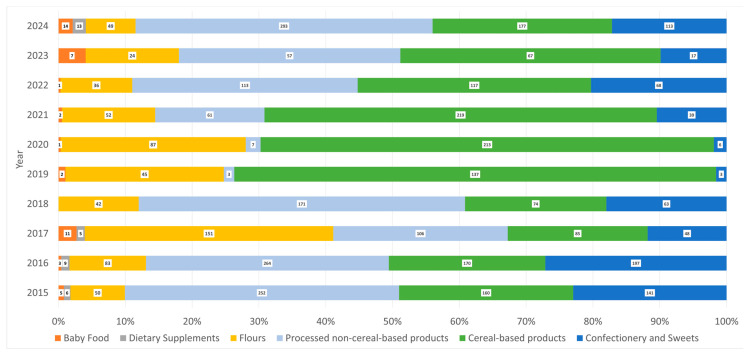
Annual composition of the dataset, showing the percentage contribution of each macro-category to the total number of samples analyzed per year.

To explore potential temporal variation, the annual proportion of non-compliant samples was calculated, and a linear regression model was fitted to assess trends over the study period (Figure 2).

**Figure 3:**
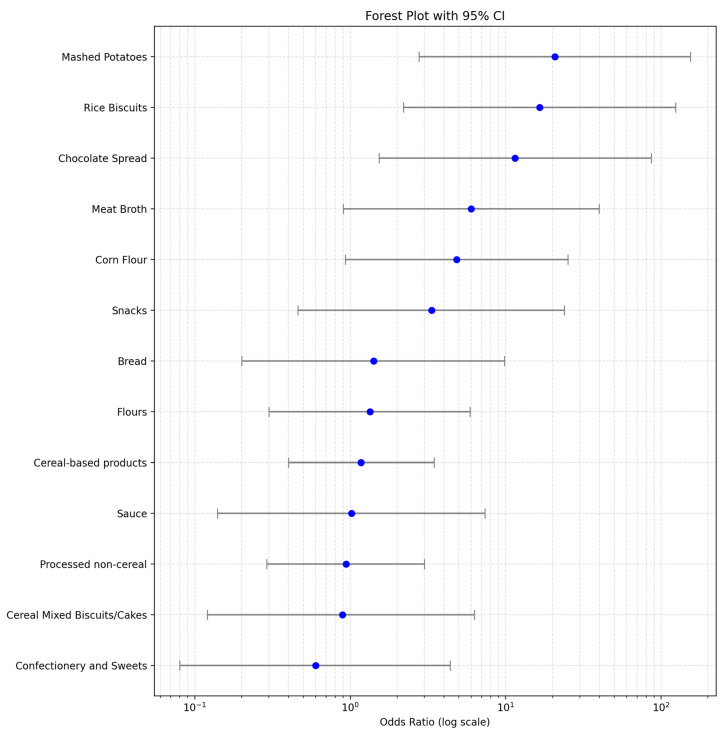
Forest plot of odds ratios (OR) and 95% confidence intervals for each food matrix compared with the overall dataset as the reference population. OR values are displayed on a logarithmic scale. Matrices with higher OR exhibit a greater relative likelihood of non-compliance, whereas matrices with OR = 0 had no observed non-compliant samples (OR values are derived from the frequency of non-compliant results, not from quantitative gluten levels).

To investigate factors associated with non-compliance, a multivariable logistic regression model was fitted. The dependent variable was binary (0 = compliant; 1 = non-compliant). Explanatory variables included the macro-category and the year of analysis (modeled as a continuous variable). Adjusted odds ratios (ORs) and their 95% confidence intervals (CIs) were calculated. For matrices with zero non-compliant samples, ORs were set to 0. A forest plot on a logarithmic scale was generated to illustrate the relative likelihood of non-compliance across matrices (Figure 3).

All analyses were performed using standard parametric statistical methods. Descriptive and graphical summaries were generated in Excel^®^ (Microsoft, Beaverton, OR, USA), while regression modeling and inferential analyses were conducted using Python 3.12.4 (packaged by Anaconda, Inc., Austin, TX, USA).

## 3. Results and Discussion

The six macro-categories used in this study (baby food, flours, processed non-cereal-based products, cereal-based products, confectionery and sweets, dietary Supplements) are further subdivided into product types as detailed in Figure 1 and Table S1 in the Supplementary Material.

The descriptive analysis of the product distribution within the food sector reveals a marked predominance of products classified as cereal-based and processed non-cereal-based products, which constitute 34% and 32% of the total sample, respectively. This is followed by confectionery and sweets and flours, representing 17% and 15%, respectively, of all the analyzed products. Baby food and dietary Supplements, as minor categories, each account for only 1% of the total sample. Within each macro-category, the most represented product types are mixed biscuits/cakes and cereal mixed pasta (cereal-based), sauces and dairy products (processed non-cereal-based), and rice and corn flours (flours) (Table S1). Baby food samples mainly include fruit-based, cereal-based, and meat-based products (Table S1).

Sample numbers varied across years, with the proportion of each macro-category remaining broadly consistent throughout the study period (Figure 2 and Table S1).

**Figure 4:**
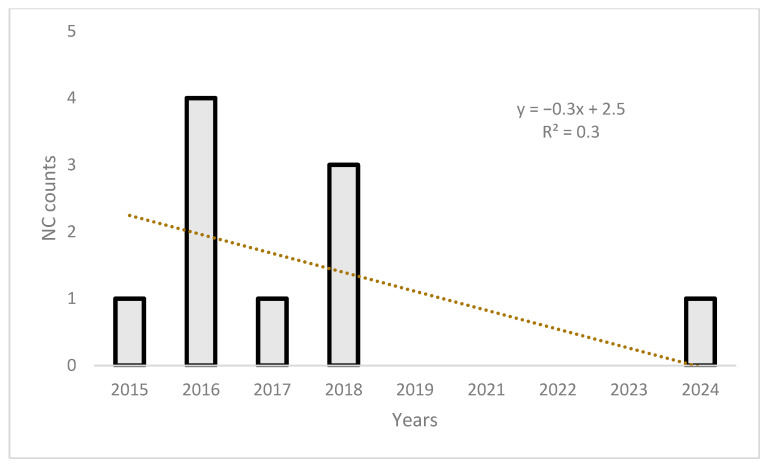
Column chart showing the annual proportion of non-compliant (NC) samples over the study period. The *Y*-axis represents the ratio of non-compliant samples (NC), while the *X*-axis represents the sampling year. The linear regression trend line indicates an overall negative trend, with the highest NC counts observed in 2016 and 2018, followed by consistently low or zero values in the subsequent years.

Notably, the proportion of non-compliant results remains relatively very low and comparable across all years and macro-categories, with isolated spikes such as in 2016 (4 non-compliant results) and 2018 (3 non-compliant results). The overall compliance rate exceeded 99% across all sample types (Figure 4; Tables S1a and S1b).

The non-compliant trend is reported in Figure 4. A linear regression trend indicates an overall negative temporal trend, driven by the isolated peaks in 2016 and 2018.

The non-compliant observed in cereal-based products (*n* = 4), flours (*n* = 2), processed non-cereal-based products (*n* = 3) and confectionery and sweets (*n* = 1) indicates possible cross-contact during processing. In cereal-based products, NC samples involved mixed bread, biscuits/cakes, rice biscuits and snacks; in Flours, all NC samples corresponded to corn flour (Table S1).

These products are staple foods and may be more exposed to gluten contamination due to their raw material sources and specific production chain [23,24]. Naturally gluten-free raw materials, such as corn flour, can become contaminated with gluten-containing cereals at various stages, including during cultivation, storage, transportation, or food production [23]. These raw materials could expose patients with CD to a possible risk for their health, as they are basic ingredients of many gluten-free staple foods. Recent research has shown that foods derived from naturally gluten-free ingredients can often contain significant levels of gluten contamination [23]. A pilot study, reported in the literature, found gluten levels as high as 2925 mg/kg in certain naturally gluten-free grains and seeds used in gluten-free food production. Additionally, flours derived from naturally gluten-free cereals have also been found to be contaminated with gluten [24]. As a result, naturally gluten-free foods or foods derived from them, if not well monitored, may present a relevant source of unintentional lapses in adherence to a gluten-free diet, which are challenging to prevent [24].

Non-compliant results within processed non-cereal-based products macro-category have been detected in mashed potatoes mix, meat broth and sauce, while one NC chocolate spread was identified within confectionery and sweets.

Such findings may be consistent with known risks of cross-contact in multi-ingredient or shared production lines [5,23,25]. Indeed, it is crucial to carefully apply validated protocols for production, cleaning and control of finished products to establish effective control measures for the presence of allergens in food [26].

The absence of non-compliant results in baby food is a comforting result, given the vulnerable consumer base [23,24], probably due to the stricter legal framework and higher controls. Indeed, the presence of gluten in baby food, albeit in low instances, could represent a serious risk for toddlers with coeliac disease or wheat allergies [23,24].

Risk modeling highlighted the highest risk for mashed potatoes (OR = 20.65, 95% CI = 2.76–154.42), rice biscuits (OR = 16.52, 95% CI = 2.20–123.55), and chocolate spread (OR = 11.49, 95% CI = 1.53–86.13). For the other matrices, the model reports a lower risk even if CIs are wide due to low event counts (Figure 3 and Table S2).

Although the dataset used in this study provides valuable information on compliance patterns across food matrices, the absence of several key variables—such as production year, manufacturer, origin of raw materials, processing conditions, and analytical parameters—limits the possibility of conducting a more detailed multivariate or causal analysis. Nevertheless, the observed distribution of odds ratios offers useful insights into potential underlying factors that may influence the likelihood of non-compliance. Several plausible hypotheses emerge from the matrix-specific risk patterns.

Matrices with higher OR values—such as mashed potato products, rice biscuits, and chocolate spreads—may indicate higher susceptibility to inadvertent gluten contamination. Regarding the mashed potatoes mix, the dilution occurring during preparation of the final product may result in diluted gluten levels in the final preparation, although the product remains non-compliant according to the regulation. However, the product remains non-compliant according to the regulation, as EU Regulation 828/2014 requires. Indeed, all these product categories often share features that may contribute to gluten introduction during manufacturing. For instance, many of them rely on multi-ingredient formulations or processing environments where both gluten-free and gluten-containing products may be handled, increasing the probability of cross-contact during mixing, weighing, flavoring, or packaging operations. In addition, the use of dry ingredients or powdered additives could increase the chance of airborne or dust-mediated contamination, especially in facilities that also process cereals or baked goods [17,27].

Starch-based matrices such as rice-derived products and potato preparations frequently originate from supply chains where shared agricultural, milling, or storage equipment is used for both gluten-free and gluten-containing raw materials. This may result in residual gluten carryover even when the final product is not intended to contain gluten [8,28]. Similarly, chocolate spreads and other composite foods often incorporate ingredients from global multi-step supply chains (e.g., cocoa, vegetable oils, flavorings), where heterogeneous production standards and shared processing lines could occasionally introduce gluten traces [8,28].

Conversely, matrices presenting lower OR values—such as many confectionery items or simpler formulations—often undergo more controlled or ingredient-restricted production, reducing opportunities for gluten ingress. These products may rely on highly refined raw materials, have fewer powder-based or cereal-derived components, or be manufactured in lines dedicated to allergen-controlled production [8,28].

Overall, the OR patterns observed in the dataset appear to reflect differences in cross-contamination susceptibility along the production and supply chain, driven by ingredient complexity, shared equipment, and the likelihood of gluten exposure during manufacturing, rather than biochemical or microbiological properties of the matrices.

These patterns reflect differences in cross-contamination susceptibility along ingredient and processing chains, rather than intrinsic matrix properties [29]. Verma et al., in an Italian study, reported that 29 of the 47 analyzed food samples—including those labeled as ‘containing gluten’, ‘naturally gluten-free’, ‘gluten-free’, or ‘may contain traces of gluten’—were found to be compliant [19]. Rasmussen et al., in their Danish study investigating products bearing precautionary allergen labeling, reported that more than 96% (i.e., 3 samples out of 77 total samples) of the samples complied with the threshold limit [30]. Gélinas et al. conducted a study in Canada to assess gluten contamination in cereal-based foods, both with and without gluten-free labeling. Among the 148 products analyzed—about half of which were labeled gluten-free—they found that 15% contained more than 20 ppm of gluten, as determined by the R5 enzyme-linked immunosorbent assay (ELISA) [27]. In the USA, 75% of gluten-free labeled oat samples were contaminated [24]. Farage et al. investigated gluten contamination in naturally gluten-free meals from 60 food establishments in the Federal District of Brazil, analyzing 180 samples using an immunoenzymatic assay with a 20 ppm gluten threshold and found that 2.8% of samples exceeded this limit [31]. In addition, they highlight the lack of consistent gluten cross-contamination controls in Brazil, where legislation neither mandates contamination prevention in food services nor establishes gluten limits for gluten-free products. Rodríguez et al., analyzing a geographically broader sample—including products from Chile as well as imports from the USA, Italy, Argentina, and Spain—found that only 64% of the gluten-free labeled samples were compliant [29].

Overall, our findings confirm very high compliance with gluten-free requirements and suggest that current control measures implemented in Italy effectively minimize the risk of gluten contamination. Regulatory frameworks, industry protocols and consumer-facing quality standards (including AOECS guidance) likely contribute to this positive performance.

Lastly, these findings highlight the importance of consumer education and transparent labeling [24]. While regulatory compliance is a crucial factor, empowering consumers with knowledge about potential gluten sources and safe consumption practices is equally critical [4]. Clear and accurate allergen labeling, coupled with public awareness campaigns, can significantly enhance the quality of life for individuals with coeliac disease, NCGS/WS, or wheat allergies [4].

## 4. Conclusions

This study shows very high compliance with EU Regulation 828/2014 and confirms the overall reliability of gluten-free labeling in Italy. A slight reduction in non-compliance over time suggests progressive improvement in industry control measures. Risk modeling identified a limited number of matrices—mashed potatoes, rice biscuits, chocolate spread, corn flour, meat broth, and snacks—as comparatively more prone to gluten contamination, indicating areas where targeted monitoring may be beneficial. Baby food and dietary supplements showed no non-compliance.

Limitations include the exclusive use of official control data, the absence of detailed production-process metadata, and the lack of systematic information on labeling status, preventing causal inference.

Future work should integrate richer metadata and harmonized sampling to enable direct comparisons between labeled and non-labeled products and more granular risk evaluation.

## Data Availability

Data are available in the Supplementary Materials.

## Supplementary Materials

The following supporting information can be downloaded at: https://www.mdpi.com/article/10.3390/foods15071144/s1, Table S1a: Annual number of compliant samples across macro-categories and sample types, along with rates relative to the total number of samples. Table S1b: Annual number of non-compliant samples across macro-categories and sample types, along with rates relative to the total number of samples. Table S2: Odds Ratios (OR) and 95% confidence intervals (CI) for non‑compliance across food matrices, calculated using the total dataset as the reference population. OR values greater than 1 indicate a higher likelihood of non‑compliance compared with the overall dataset, whereas OR = 0 reflects matrices with no observed non‑compliant samples.

## Abbreviations

The following abbreviations are used in this manuscript:

CD	Coeliac disease
NCGS/WS	Non-coeliac gluten sensitivity/wheat sensitivity
TM	Trademark
PAL	Precautionary Allergen Labeling
AOECS	Association Of European Coeliac Societies
C	Compliant, gluten concentration ≤ 20 ppm
NC	Non-compliant, gluten concentration > 20 ppm
