# Supplementary material for: Ensuring Gluten-Free Safety: A Descriptive Analysis of Laboratory Results and Quality Control
Source: Foods. 2026 Mar 26;15(7):1144. doi: 10.3390/foods15071144 (PMC13073890; doi:10.3390/foods15071144)
Supplement: Supplementary file 1 [file foods-15-01144-s001.zip › foods-4141565-supplementary.pdf]

Table S1a: Annual number of compliant samples across macro-categories and sample types, along with rates relative to the total number of samples.

Macro-categories/Sample Size	Year																				Total	
	2015		2016		2017		2018		2019		2020		2021		2022		2023		2024			
	n°	%	n°	%	n°	%	n°	%	n°	%	n°	%	n°	%	n°	%	n°	%	n°	%	n°	%
**Baby Food**	**5**	**0,82**	**3**	**0,42**	**11**	**2,72**		**0**	**2**	**1,05**	**1**	**0,32**	**2**	**0,54**	**1**	**0,3**	**7**	**4,07**	**14**	**2,13**	**46**	**1,11**
Baby Chamomile	0	0	1	0,14	0	0	0	0	0	0	0	0	0	0	0	0	0	0	1	0,15	**1**	**0,02**
Baby Food Cereal	0	0	0	0	5	1,23	2	0,58	1	0,53	0	0	1	0,27	1	0,3	4	2,33	14	2,13	14	0,34
Baby Food Cod	0	0	0	0	1	0,25	0	0	0	0	0	0	0	0	0	0	0	0	1	0,15	1	0,02
Baby Food flavoured/mixed with other ingredients diary	0	0	0	0	0	0	0	0	0	0	0	0	0	0	0	0	2	1,16	2	0,3	2	0,05
Baby Food Fruit	1	0,16	1	0,14	2	0,49	0	0	0	0	2	0,64	0	0	5	1,49	4	2,33	15	2,28	15	0,36
Baby Food Meat	1	0,16	1	0,14	3	0,74	0	0	0	0	0	0	0	0	1	0,3	4	2,33	10	1,52	10	0,24
Baby Food Vegetable	3	0,49	0	0	0	0	0	0	0	0	0	0	0	0	0	0	0	0	3	0,46	**3**	**0,07**
**Dietary Supplements**	**6**	**0,98**	**9**	**1,25**	**5**	**1,23**	**0**	**0**	**0**	**0**	**0**	**0**	**0**	**0**	**0**	**0**	**0**	**0**	**13**	**1,98**	**33**	**0,8**
**Flours**	**50**	**8,16**	**81**	**11,22**	**151**	**37,28**	**42**	**12,1**	**45**	**23,68**	**87**	**27,71**	**52**	**13,94**	**36**	**10,75**	**24**	**13,95**	**49**	**7,45**	**617**	**14,94**
Amaranth Flour	0	0	0	0	0	0	0	0	0	0	0	0	1	0,27	0	0	0	0	0	0	1	0,02
Brown Millet Flour	0	0	0	0	0	0	1	0,29	0	0	0	0	0	0	1	0,3	0	0	0	0	2	0,05
Buckwheat Flour	5	0,82	3	0,42	2	0,49	3	0,86	1	0,53	7	2,23	1	0,27	0	0	1	0,58	1	0,15	24	0,58
Corn Flour	20	3,26	29	4,02	16	3,95	16	4,61	17	8,95	34	10,83	10	2,68	12	3,58	4	2,33	13	1,98	171	4,14
Millet Flour	0	0	0	0	0	0	0	0	0	0	0	0	1	0,27	0	0	0	0	0	0	1	0,02
Mixed Flour	1	0,16	20	2,77	3	0,74	2	0,58	7	3,68	7	2,23	6	1,61	0	0	0	0	0	0	46	1,11
Oat Flour	0	0	0	0	0	0	0	0	0	0	0	0	0	0	0	0	1	0,58	1	0,15	2	0,05
Quinoa Flour	0	0	1	0,14	0	0	0	0	0	0	0	0	1	0,27	0	0	0	0	0	0	2	0,05
Rice Flour	24	3,92	28	3,88	128	31,6	18	5,19	19	10	39	12,42	28	7,51	23	6,87	18	10,47	34	5,17	359	8,69
Sorghum Flour	0	0	0	0	1	0,25	0	0	0	0	0	0	1	0,27	0	0	0	0	0	0	2	0,05
Teff Flour	0	0	0	0	1	0,25	0	0	1	0,53	0	0	3	0,8	0	0	0	0	0	0	5	0,12
Whole Sorghum Flour	0	0	0	0	0	0	2	0,58	0	0	0	0	0	0	0	0	0	0	0	0	2	0,05
**Processed non-cereal-based products**	**251**	**40,95**	**264**	**36,57**	**106**	**26,17**	**169**	**48,7**	**3**	**1,58**	**7**	**2,23**	**61**	**16,35**	**113**	**33,73**	**57**	**33,14**	**293**	**44,53**	**1324**	**32,07**
Cake Colouring/Flavouring	1	0,16	7	0,97	0	0	0	0	0	0	0	0	0	0	0	0	0	0	1	0,15	9	0,22

Carob Powder	0	0	0	0	1	0,25	0	0	0	0	0	0	0	0	0	0	0	0	0	0	1	0,02
Carot Powder	0	0	1	0,14	0	0	0	0	0	0	0	0	0	0	0	0	0	0	0	0	1	0,02
Chestnuts/Hazelnuts Powder	2	0,33	10	1,39	1	0,25	2	0,58	0	0	0	0	1	0,27	1	0,3	0	0	2	0,3	19	0,46
Chickpea/Pea/Soy Powder	4	0,65	5	0,69	5	1,23	6	1,73	0	0	1	0,32	5	1,34	7	2,09	3	1,74	11	1,67	47	1,14
Chocolate Drink	1	0,16	1	0,14	1	0,25	0	0	0	0	0	0	0	0	0	0	0	0	0	0	3	0,07
Cocoa Powder	2	0,33	0	0	0	0	0	0	0	0	0	0	0	0	0	0	0	0	8	1,22	10	0,24
Coffee Drink	11	1,79	3	0,42	2	0,49	0	0	0	0	0	0	1	0,27	4	1,19	0	0	13	1,98	34	0,82
Desiccated Coconut	5	0,82	3	0,42	2	0,49	1	0,29	0	0	0	0	1	0,27	1	0,3	0	0	3	0,46	16	0,39
Digestive Tablets	1	0,16	2	0,28	0	0	0	0	0	0	0	0	0	0	1	0,3	0	0	0	0	4	0,1
Energy Drink	2	0,33	0	0	1	0,25	0	0	0	0	0	0	1	0,27	0	0	0	0	0	0	4	0,1
Flavoured/mixed with other ingredients diary	52	8,48	49	6,79	7	1,73	49	14,12	1	0,53	2	0,64	10	2,68	15	4,48	8	4,65	107	16,26	300	7,27
Fruit Drink	23	3,75	13	1,8	17	4,2	9	2,59	2	1,05	0	0	7	1,88	17	5,07	3	1,74	12	1,82	103	2,49
Fruit Ice Cream	1	0,16	10	1,39	0	0	11	3,17	0	0	0	0	0	0	0	0	0	0	0	0	22	0,53
Fruit Jam	33	5,38	28	3,88	13	3,21	22	6,34	0	0	0	0	0	0	0	0	0	0	3	0,46	99	2,4
Gaseous Drink	0	0	0	0	1	0,25	0	0	0	0	0	0	0	0	0	0	0	0	0	0	1	0,02
Gelling agent	0	0	7	0,97	2	0,49	0	0	0	0	0	0	0	0	1	0,3	0	0	2	0,3	12	0,29
Herbs Drink	1	0,16	1	0,14	0	0	2	0,58	0	0	1	0,32	0	0	2	0,6	0	0	7	1,06	14	0,34
Herbs Powder	4	0,65	2	0,28	6	1,48	0	0	0	0	1	0,32	0	0	0	0	1	0,58	0	0	14	0,34
Mashed Potatoes Mix	6	0,98	7	0,97	2	0,49	2	0,58	0	0	0	0	1	0,27	2	0,6	0	0	0	0	20	0,48
Meat Broth	15	2,45	13	1,8	0	0	11	3,17	0	0	0	0	0	0	16	4,78	6	3,49	8	1,22	69	1,67
Potato Pasta	0	0	1	0,14	0	0	0	0	0	0	0	0	1	0,27	0	0	0	0	0	0	2	0,05
Potato/Tapioca Powder	2	0,33	2	0,28	1	0,25	3	0,86	0	0	1	0,32	2	0,54	4	1,19	0	0	6	0,91	21	0,51
Processed Vegetables	5	0,82	8	1,11	3	0,74	7	2,02	0	0	0	0	1	0,27	8	2,39	12	6,98	33	5,02	77	1,86
Sauce	80	13,05	84	11,63	40	9,88	42	12,1	0	0	1	0,32	30	8,04	34	10,15	24	13,95	70	10,64	405	9,81
Tempeh/Tofu	0	0	0	0	0	0	0	0	0	0	0	0	0	0	0	0	0	0	5	0,76	5	0,12
Vegetable Broth	0	0	0	0	1	0,25	1	0,29	0	0	0	0	0	0	0	0	0	0	0	0	2	0,05
Vinegar	0	0	7	0,97	0	0	1	0,29	0	0	0	0	0	0	0	0	0	0	2	0,3	10	0,24
**Cereal-based products**	**160**	**26,1**	**168**	**23,27**	**84**	**20,74**	**73**	**21,04**	**137**	**72,11**	**213**	**67,83**	**219**	**58,71**	**117**	**34,93**	**67**	**38,95**	**177**	**26,9**	**1415**	**34,27**
Bread	15	2,45	19	2,63	18	4,44	19	5,48	43	22,63	61	19,43	58	15,55	24	7,16	10	5,81	25	3,8	292	7,07

Buckwheat Cakes	0	0	3	0,42	0	0	0	0	1	0,53	6	1,91	4	1,07	2	0,6	2	1,16	5	0,76	23	0,56
Cereal Drink	28	4,57	9	1,25	2	0,49	8	2,31	2	1,05	7	2,23	4	1,07	4	1,19	7	4,07	32	4,86	103	2,49
Cereal Mixed Biscuits/Cakes	35	5,71	55	7,62	29	7,16	12	3,46	55	28,95	67	21,34	86	23,06	55	16,42	26	15,12	46	6,99	466	11,29
Cereal Mixed Pasta	30	4,89	14	1,94	9	2,22	4	1,15	29	15,26	46	14,65	39	10,46	11	3,28	9	5,23	12	1,82	203	4,92
Corn Biscuits/Cakes/Flakes	7	1,14	3	0,42	5	1,23	2	0,58	3	1,58	4	1,27	7	1,88	0	0	0	0	3	0,46	34	0,82
Pudding	28	4,57	36	4,99	10	2,47	19	5,48	1	0,53	1	0,32	4	1,07	6	1,79	8	4,65	22	3,34	135	3,27
Quinoa Biscuits	0	0	0	0	0	0	0	0	1	0,53	1	0,32	1	0,27	1	0,3	0	0	2	0,3	6	0,15
Rice Biscuits	4	0,65	1	0,14	0	0	1	0,29	0	0	12	3,82	7	1,88	0	0	0	0	0	0	25	0,61
Rice ready to eat	0	0	0	0	0	0	0	0	0	0	4	1,27	0	0	0	0	0	0	0	0	4	0,1
Snacks	13	2,12	28	3,88	11	2,72	8	2,31	2	1,05	4	1,27	9	2,41	14	4,18	5	2,91	30	4,56	124	3
**Confectionery and Sweets**	**141**	**23**	**197**	**27,29**	**48**	**11,85**	**63**	**18,16**	**3**	**1,58**	**6**	**1,91**	**39**	**10,46**	**68**	**20,3**	**17**	**9,88**	**112**	**17,02**	**694**	**16,81**
Almond/Hazelnuts/Peanuts/Pistachio Spread	0	0	0	0	0	0	0	0	0	0	0	0	0	0	0	0	0	0	8	1,22	8	0,19
Candy	64	10,44	66	9,14	24	5,93	23	6,63	0	0	1	0,32	12	3,22	26	7,76	4	2,33	53	8,05	273	6,61
Chocolate Bar	66	10,77	93	12,88	18	4,44	33	9,51	1	0,53	1	0,32	16	4,29	40	11,94	10	5,81	23	3,5	301	7,29
Chocolate Spread	5	0,82	11	1,52	0	0	3	0,86	0	0	0	0	0	0	1	0,3	0	0	16	2,43	36	0,87
Fruit Syrup	0	0	6	0,83	1	0,25	1	0,29	2	1,05	4	1,27	11	2,95	0	0	2	1,16	6	0,91	33	0,8
Sugar/Sugar Paste/Sweetener	6	0,98	19	2,63	5	1,23	3	0,86	0	0	0	0	0	0	1	0,3	1	0,58	6	0,91	41	0,99
Vanilla Spread	0	0	2	0,28	0	0	0	0	0	0	0	0	0	0	0	0	0	0	0	0	2	0,05
**Totale complessivo**	**613**	**100**	**722**	**100**	**405**	**100**	**347**	**100**	**190**	**100**	**314**	**100**	**373**	**100**	**335**	**100**	**172**	**100**	**658**	**100**	**4129**	**100**

Table S1b: Annual number of non-compliant samples across macro-categories and sample types, along with rates relative to the total number of samples.

Macro-categories/Sample Size	Year										Totale complessivo	
	2015		2016		2017		2018		2024			
	n°	%	n°	%	n°	%	n°	%	n°	%	n°	%
**Flours**	**0**	**0**	**2**	**50**	**0**	**0**	**0**	**0**	**0**	**0**	**2**	**20**
Corn Flour	0	**0**	2	50	0	0	0	0	0	0	2	20
**Processed non-cereal-based products**	**1**	**100**	**0**	**0**	**0**	**0**	**2**	**66,7**	**0**	**0**	**3**	**30**
Mashed Potatoes	0	**0**	0	0	0	0	1	33,3	0	0	1	10
Meat Broth	0	**0**	0	0	0	0	1	33,3	0	0	1	10
Sauce	1	**100**	0	0	0	0	0	0	0	0	1	10
**Cereal-based products**	**0**	**0**	**2**	**50**	**1**	**100**	**1**	**33,3**	**0**	**0**	**4**	**40**
Bread	0	**0**	0	0	1	100	0	0	0	0	1	10
Cereal Mixed Biscuits/Cakes	0	**0**	1	25	0	0	0	0	0	0	1	10
Rice Biscuits	0	**0**	1	25	0	0	0	0	0	0	1	10
Snacks	0	**0**	0	0	0	0	1	33,3	0	0	1	10
**Confectionery and Sweets**	**0**	**0**	**0**	**0**	**0**	**0**	**0**	**0**	**1**	**100**	**1**	**10**
Chocolate Spread	0	**0**	0	0	0	0	0	0	1	100	1	10
**Totale complessivo**	**1**	**100**	**4**	**100**	**1**	**10**	**3**	**100**	**1**	**100**	**10**	**100**

Table S2: Odds Ratios (OR) and 95% confidence intervals (CI) for non-compliance across food matrices, calculated using the total dataset as the reference population. OR values greater than 1 indicate a higher likelihood of non-compliance compared with the overall dataset, whereas OR = 0 reflects matrices with no observed non-compliant samples.

Matrix	OR	Lower CI	Upper CI
Confectionery and Sweets	0.60	0.08	4.38
Cereal Mixed Biscuits/Cakes	0.89	0.12	6.30

Processed non-cereal	0.94	0.29	3.00
Sauce	1.02	0.14	7.33
Cereal-based products	1.17	0.40	3.47
Flours	1.34	0.30	5.91
Bread	1.41	0.20	9.83
Snacks	3.33	0.46	23.84
Corn Flour	4.83	0.93	25.10
Meat Broth	6.00	0.90	39.80
Chocolate Spread	11.49	1.53	86.13
Rice Biscuits	16.52	2.20	123.55
Mashed Potatoes	20.65	2.76	154.42
